# Motor reaction time in older women with different degrees of sarcopenia: What do electroencephalograms show?

**DOI:** 10.31744/einstein_journal/2026AO1541

**Published:** 2026-05-21

**Authors:** Raquel Brito Elmescany, Iully Ingrid Pereira da Silva Alves, Guilherme Augusto Santos Bueno, Silvana Schwerz Funghetto, Juliana de Faria Fracon e Romão, Valéria Pagotto, Anabela Correia Martins, Ruth Losada de Menezes

**Affiliations:** 1 Universidade Federal de Goiás Postgraduate Program in Health Sciences Faculdade de Medicina Goiânia GO Brazil Postgraduate Program in Health Sciences, Faculdade de Medicina, Universidade Federal de Goiás, Goiânia, GO, Brazil.; 2 Universidade de Brasília Campus of Ceilândia Postgraduate Program in Health Sciences and Technologies Brasília DF Brazil Postgraduate Program in Health Sciences and Technologies, Campus of Ceilândia, Universidade de Brasília, Brasília, DF, Brazil.; 3 Universidade de Brasília Campus of Ceilandia Brasília DF Brazil Campus of Ceilandia, Universidade de Brasília, Brasília, DF, Brazil.; 4 Universidade Federal de Goiás Escola de Enfermagem Postgraduate Program in Nursing Goiânia GO Brazil Postgraduate Program in Nursing, Escola de Enfermagem, Universidade Federal de Goiás, Goiânia, GO, Brazil.; 5 Polytechnic Institute of Coimbra College of Health Technology of Coimbra Coimbra Portugal College of Health Technology of Coimbra, Polytechnic Institute of Coimbra, Coimbra, Portugal.

**Keywords:** Sarcopenia, Motor skills, Reaction time, Electroencephalography, Brain mapping, Geriatric, Age

## Abstract

**Objective:**

This study aimed to analyze cortical activity during simple motor reaction time tasks in older women with different degrees of sarcopenia.

**Methods:**

This cross-sectional, observational, analytical study included 59 women classified into control, pre-sarcopenia, and sarcopenia groups according to European Working Group on Sarcopenia in Older People criteria. Motor reaction time was assessed through standardized visual stimuli with simultaneous electroencephalography. All participants received identical instructions and completed a practice block. The trials were verified by blinded raters. Electroencephalogram alpha (8-12 Hz) and beta (12-28 Hz) power ratios were analyzed as markers of cortical excitation using a false discovery rate correction (q=0.05).

**Results:**

Pre-sarcopenic participants exhibited significantly higher cortical excitation than did the control and sarcopenic groups (p<0.001), suggesting that compensatory hyperactivation precedes cortical decline.

**Conclusion:**

Sarcopenia involves central neurophysiological changes in addition to muscle atrophy. The pre-sarcopenic stage represents an optimal window for neuromotor intervention given its increased cortical responsiveness.

## In Brief

Cortical activity during motor tasks differed across sarcopenia stages. EEG showed increased cortical excitation in presarcopenia, suggesting compensatory hyperactivation, followed by reduced responsiveness to sarcopenia. These findings highlight the role of the brain-muscle connection in aging.

## Highlights

■ Pre-sarcopenic women showed increased cortical excitation during motor tasks.
■ Sarcopenia was associated with slower motor reaction time.
■ EEG revealed central nervous system changes across sarcopenia stages.
■ Pre-sarcopenia may represent a window for early neuromotor intervention.

## Graphical abstract

**Figure:**
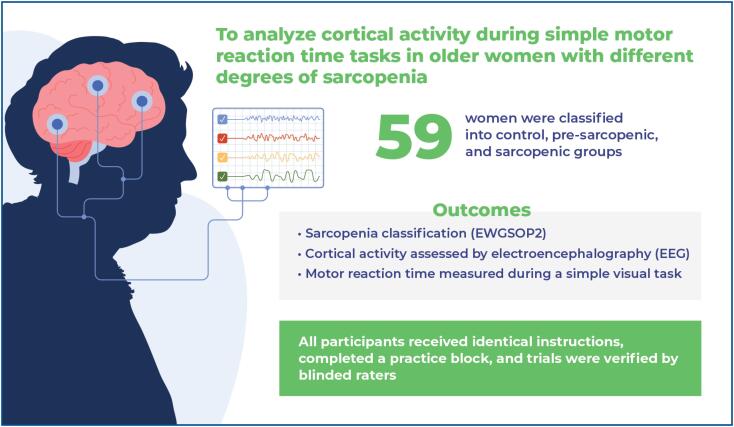

## INTRODUCTION

A continuous increase in life expectancy, especially in the older age groups, has become a prevailing trend in many countries. Consequently, population aging is now a global phenomenon that demands a deeper understanding of effective solutions to the challenges associated with the care and well-being of older individuals.^(1)^ According to a United Nations report,^(2)^ the number of individuals aged 65 and over is expected to grow significantly from 0.7 billion (9% of the global population) in 2019 to 1.5 billion (16%) in 2050. Furthermore, recent studies^(3)^ have indicated that population aging will be accompanied by a 55% increase in disability-adjusted life years in people aged over 60 years between 2004 and 2030.

Notably, changes in body composition due to aging do not follow a linear trajectory and do not affect men or women in the same manner. After menopause, hormonal fluctuations, including decreased estrogen and testosterone levels, result in the loss of muscle quality and reduced strength in women, especially in the lower limbs.^(4,5)^ However, regardless of sex, researchers^(6)^ suggest that the loss of muscle quality with aging may be the result of several factors and adaptations related to the nervous system, including decreased efficiency of the cerebral cortex, spinal cord, and neuromuscular connections. Therefore, exploring the role of these factors is crucial to better understand the reasons for muscle function deterioration in older adults.

Aging-related supraspinal adaptations have been observed in the motor cortical regions of the frontal lobe, which houses a variety of excitatory and inhibitory neurons, including glutaminergic neurons which are involved in neural development, synaptic plasticity, learning, and memory, and play a fundamental role in the mechanism of some neurodegenerative diseases.^(7)^ Several studies^(8,9)^ have highlighted cortical atrophy in these regions, with a decrease in the total volume of these regions, ranging from 4%-16%, throughout the aging process. These adaptations observed in the primary motor cortex appear to be associated with a reduction in the size of motor and premotor neurons, as well as a reduction in synaptic transmission. This phenomenon is known as neural atrophy.^(8)^ A study using dissections in cadavers revealed that older adults present, on average, a 43% reduction in the size of motor cortex cells compared with that in young adults.^(10)^ Moreover, another study^(11)^ of cadavers carried out at the University of São Paulo revealed that brain weight and volume decreased during aging by approximately 45 g and 43 mL per decade, respectively, and these reductions were more pronounced in women. These findings have now been corroborated by recent studies employing high-resolution magnetic resonance imaging in living subjects. In a 15-year cohort study, Fujita et al.^(11)^ reported an annual decrease of -0.5% of the cerebral cortex; the frontal lobe showed a great dependence on age (annual decrease in volume -0.5%) and was related with morphological changes in neural cells. Other motor functions, such as a decline in the motor performance of the lower limbs^(13)^ and gait, also appear to be affected by neural atrophy.^(14)^ A meta-analysis of brain imaging studies addressed the effects of aging on motor control and described a behavioral decline in older participants, suggesting a general deterioration in motor functions associated with the physiological process of aging; older adults showed increased reaction times (five studies), reduced precision (12 studies), or both (four studies) during the execution of different motor tasks.^(15)^

However, neural atrophy is not the only factor associated with aging as significant changes also occur in the cortical white matter of individuals aged >65 years.^(16,17)^ This region, which is composed mainly of glial cells and myelinated axons, is crucial for the connectivity between different cortical areas and the spinal cord. A previous study^(12)^ reported considerable deterioration in the integrity of white matter with an age-related decrease in volume and an accelerated rate of atrophy. Additionally, experimental research^(18,19)^ has revealed a reduction in the length of myelinated nerve fibers in the white matter of older individuals compared to that in younger individuals. These age-related morphological changes may contribute to a state of disconnection between cortical areas, which is associated with declines in executive function in both fine and gross motor skills and in information processing speed.^(18,20)^ Furthermore, evidence indicates that changes in white matter may lead to reduced stability, manifested by greater oscillation in the trajectory and impairment of static balance.^(19)^

Dysfunction of the glutamatergic system is one of the neurochemical mechanisms underlying this increase in neural noise, because glutamate plays a central role in modulating excitatory signals in the central nervous system. Accordingly, the significant reduction in glutamate absorption caused by aging, which leads to an increase in the glutamate concentration around cortical neurons, has been associated with increased neural noise.^(21)^ Additionally, neuromuscular decline, which involves several physiological processes that affect the nervous and muscular systems, such as loss of motor neurons, is associated with aging. Aging decreases the number of motor neurons in the spinal cord that are responsible for sending nerve signals to the muscles. This can lead to a reduction in the ability to recruit motor units, which are groups of muscle fibers controlled by motor neurons.^(22)^ The speed at which these signals are transmitted by peripheral nerves also tends to decrease because of structural changes in the motor endplate, where the connection between the motor neuron and the muscle fiber occurs.^(23)^ Furthermore, muscle fibers tend to change, resulting in a transition from fast- to slower-twitch fibers, which affects muscle speed and strength.^(24)^ Several studies have reported that the ability of muscles to regenerate after injury or exercise can also reduce with age, owing to changes in the cellular and molecular environment.^(25)^ This reduction may be related to failures in the denervation and reinnervation processes, which normally help compensate for neuronal loss and consequent problems with muscle strength and control. Fiber denervation rates far exceed reinnervation rates, and a decrease in fiber size is directly linked to increased oxidative stress and cellular apoptosis, resulting in a significant reduction in the number of satellite cells responsible for muscle regeneration.^(26)^

Despite these neuromuscular changes, conditions such as sarcopenia, another major challenge faced by many older adults, can exacerbate neural and muscular decline. According to the European Working Group on Sarcopenia in Older People (EWGSOP 2),^(26)^ sarcopenia is characterized by dynapenia, muscle atrophy, and poor physical performance. This phenomenon not only compromises the physical strength and mobility of this population, but is also closely linked to changes in the central nervous system (CNS) and a high risk of adverse health outcomes, including low survival rates, postoperative complications, and longer hospital stays in patients, as well as falls and fractures, metabolic disorders, cognitive impairment, and loss of autonomy.^(27-32)^ Given the increasing population of older adults, the prevalence of sarcopenia is expected to increase in the future. A recent study^(33)^ showed that according to the definition of prevalence estimates, the number of people affected by sarcopenia in Europe is likely to increase from 10,869,527 in 2016 to 18,735,173 in 2045, representing a 72.4% increase in the global prevalence of sarcopenia in older individuals, rising from 11.1% in 2016 to 12.9% in 2045. This growth may have considerable implications for society in terms of the demand for healthcare and associated costs.^(34)^ Despite advances in the understanding of sarcopenia, the neurophysiological mechanisms underlying motor performance across different stages of the condition remain insufficiently explored. In particular, cortical activity during motor tasks, especially in relation to motor reaction time, has not been adequately investigated in this context.

Consequently, interventions aimed at minimizing impairments associated with sarcopenia in this population are essential. However, no previous study has evaluated cortical activity patterns in relation to motor reaction time across different stages of sarcopenia in older women or explored how these responses may inform targeted rehabilitation strategies.

In this context, electroencephalography represents a promising tool for assessing brain activity, as it allows the analysis of electrical signals across different brain regions with high temporal resolution. This approach may provide important insights into activation patterns, including compensatory mechanisms in pre-sarcopenia and reduced responsiveness in sarcopenia.

Therefore, investigating these mechanisms may contribute to a better understanding of the muscle-brain interaction in aging and support the development of more effective neuromuscular rehabilitation strategies.^(34,35)^

## OBJECTIVE

This study aimed to analyze cortical activity using an electroencephalogram during the motor reaction time in older women with sarcopenia and pre-sarcopenia.

## METHODS

### Design and ethical aspects

This cross-sectional, observational, analytical study was conducted in the physiotherapy laboratory of the *Universidade de Brasília - Faculdade de Ceilândia* between January and March 2020. The study followed the methodological strategies of Strengthening the Reporting of Observational Studies in Epidemiology (STROBE).^(36)^ The study protocol was approved by the Research Ethics Committee of the *Faculdade de Ceilândia, Universidade de Brasília* (CAAE: 67284917.2.0000.8093; # 2,109,807). All eligible older women were informed about the study, had their questions clarified, and signed a Free and Informed Consent Form.

### Sample

Initially, a pilot study was conducted with 15 volunteers (five in the Control Group, five pre-sarcopenic, and five sarcopenic) to determine the required sample size. The sample size calculation was performed using G*Power 3.1.9.2 software (Franz Faul, Universitat Kiel, Germany),^(37)^ considering the intergroup variance of Simple Motor Reaction Time. The minimum sample size required to detect a significant and clinically important difference, with an effect size of 1.32, p<0.05, and a power of 0.95, was n=45 (n=15 per group).

The inclusion criteria for volunteers were as follows: older women 1) capable of standing and walking independently; 2) agreeing to participate in the study and signing the informed consent form; 3) with a body mass index (BMI)<30kg/m2; 4) preserved cognition (Mini Mental State Examination score >18 points);^(38)^ and 5) aged 60 years or older. The following individuals were excluded from the study: volunteers with 1) neurological diseases and/or sequelae; 2) vestibular system diseases; 3) uncorrected visual impairment; 4) orthopedic alterations, such as amputations, fractures, and history of ankle sprain in the last six months; 5) use of alcoholic beverages in the last 24 h prior to the evaluations; 6) report of osteoarthritis in the spine and/or endoprosthesis in the lower limbs; and 7) medical diagnosis of rheumatoid arthritis.

After invitation, screening, and complete evaluation, the study sample comprised 59 female participants. Of them, four were excluded for having a BMI >30kg/m2, one for having a Mini Mental State Examination score <18 points, and two for not performing all the tests owing to a lack of previously instructed preparation to undergo the electroencephalography. Data collection was carried out by properly trained researchers, including physiotherapists and undergraduate students in the last two years of the course. Each participant underwent the complete evaluation in approximately 1 h and 40 min.

### Instruments for diagnosis and subclassification of sarcopenia

According to the EWGSOP 2, three variables are used to diagnose sarcopenia in this study: muscle strength, muscle mass, and physical performance.^(26)^ Handgrip strength (HGS) was assessed using a Jamar® dynamometer to evaluate the muscular strength of the volunteers. Three measurements were recorded and then the highest value was used for analysis for the dominant and non-dominant hand, with a 1-min interval between each measurement. The reference values for Handgrip Strength Dynamometry in older women are as follows: Normal: ≥16kg and Low HGS (sarcopenia):<16kg.

Subsequently, the Omron Bioimpedance Platform was used to assess muscle mass to calculate body composition via the different levels of electrical conduction of biological tissues exposed to current frequencies emitted by the platform. This current runs through the body through electrodes and provides estimates of an individual's body composition, that is, the amount of fat present in the body in proportion to the body weight of the person being assessed. The EWGSOP sarcopenia cutoff point for muscle mass in women is 5.76-6.75kg/m2.

Finally, the Timed Up and Go (TUG) test^(39)^ was used to assess the physical performance, mobility, and functional balance of the volunteers, in which the older women were instructed to perform the following steps:

1. Standing-up from a chair: The participant began by sitting in a standard chair, with their feet flat on the floor and arms resting on the chair. The participant was then instructed to stand up from a chair without assistance and walk three meters in a straight line. Gait speed and safety were assessed during the test.
2. Turning: After walking three meters, the participant was instructed to turn around in a safe and controlled manner.
3. Return: The participant was instructed to walk to the chair and sit down safely.

The time spent in seconds to complete a task was scored, which allows the identification of any difficulty or slowness that may indicate changes in mobility, physical performance, or balance. The reference values for the TUG test were: up to 10 sec, normal performance and good balance; 11-19 s, slightly impaired mobility or mild balance problems; and ≥20 sec, an increased risk of falls and may suggest a significant deficit in mobility, muscle weakness, or serious balance problems as a cut-off point for sarcopenia.^(32)^

After the tests, the volunteers were subdivided into the following groups: 1) Control Group: those who presented minimal changes in the variables (n=15); 2) pre-sarcopenic group: those who presented with low muscle mass (n=19); and 3) sarcopenic group: those who presented with low muscle mass and low muscle strength or physical performance (n=18).

### Instruments for health aspects and functional skills

#### 30-Second Chair Stand Test

The 30-second chair stand test was used to assess leg strength and endurance. This test is especially useful in assessing the functional capacity of older individuals, as the ability to get up from a chair is essential for independence and quality of life. The volunteer had to perform the task of sitting down and getting up from a chair, repeating this action for 30 s while keeping her hands crossed over her chest or shoulders. The number of times she was able to perform the test was assessed; the fewer the repetitions, the greater the risk of falling.^(40)^ The cutoff score for sarcopenia was >15 s for five climbs.^(26)^

#### Falls Efficacy Scale International

The Falls Efficacy Scale International (FES-I-BR) scale^(41)^ was administered to assess the level of concern about falling in relation to each activity in the volunteers’ daily lives. Fear of falling is a common concern among older adults and can have a significant impact on their quality of life, independence, and physical function. The FES-I-BR seeks to quantify this fear through a series of items related to daily activities that may be affected by the fear of falling, such as walking on uneven surfaces, climbing stairs, and taking a bath.

The FES-I consists of 16 items and presents four possible responses, with scores from 1-4, where 1 represents "I am not afraid" and 4 represents "I am very afraid." The total score can range from 16-64, where a score of 16 corresponds to no concern and 64 corresponds to extreme concern about falling while performing specific activities.

#### 4-stage balance test

The 4-stage^(42)^ balance test was administered to evaluate the static balance of the volunteers based on their ability to maintain four different positions in an orthostatic posture, namely, with the feet side by side, with the midfoot next to the contralateral hallux, with one foot in front of the other, and standing with only one lower limb. The postures were performed without a support device, with the eyes open, and remaining in each position for 10 s. The performance at each of these stages was evaluated based on the participants ability to maintain posture without losing balance or needing external support to stabilize. Unstable performance at these stages may indicate a greater risk of falls and physical functional limitations.

#### Gait analyzer

The Gait Analyzer^(43)^ was used to quantify the degree of gait balance. The test is based on a smartphone application that measures the accelerations produced during gait movements using a mobile device camera to capture videos of patients as they walk. The application uses advanced image analysis and video processing algorithms to identify and quantify several gait-related parameters. With the cell phone attached to the waist in a belt with a pouch, the volunteer was asked to walk at a comfortable speed for a distance of at least 20m. At the end of the test, the results appeared in the application itself, showing all the gait variables (number of steps, total walking time, total distance covered, walking speed, walking cadence, step length, step time, step time symmetry, step length symmetry, and vertical displacement of the center of mass).

#### 10-meter Walk Test

The 10-meter walk test was used to evaluate the kinematic and spatiotemporal parameters of gait^(44)^ and was synchronized with the Gait Analyzer. The test consisted of a 20-m walk, with the initial 5m for acceleration and the final 5m for deceleration, which were disregarded. The volunteer was instructed to walk at her fastest walking speed using typical footwear and without running along the route, on a flat and straight surface, without obstacles. An assistive device could be used, if necessary. The time required to complete this distance was timed with a stopwatch. The EWGSOP cutoff point for sarcopenia that represents low performance in gait speed is ≤0.8m/s32.

#### Motor reaction time

**Figure 1:**
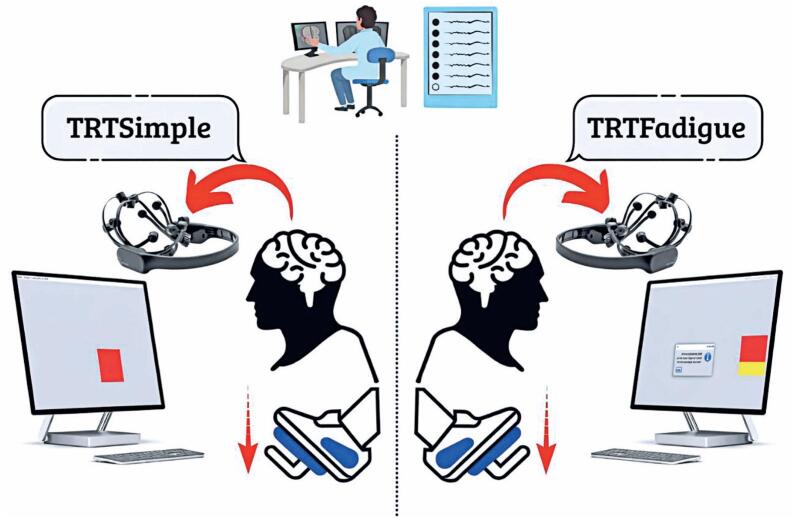
Collection of motor reaction time using electroencephalography

The motor reaction time test was used to measure the simple motor reaction time (SMRT) and fatigue reaction time (SMRT), using the TRT_S201252 Software with the adaptation of a "pedal" joystick as a command point. The participants were required to respond as quickly as possible to the stimuli generated by the software (Figure 1).

In the TRTSimple test, a red square (parameterizable) appeared in the center of the monitor at previously defined time intervals (ranging from 1.5-6.5ms; these intervals were identical in both software programs), to which the participant had to react as quickly as possible by pressing the space bar on the computer keyboard. In the TRTFadiga test, a yellow bar moved from left to right on the computer screen. The participant had to react as quickly as possible by pressing the space bar on the keyboard when the color appeared (parameterizable) and keep the key pressed, followed by the movement of the bar until it disappeared, at which point the space bar had to be released. Two TRTs were identified: TRTiFadiga for pressing the space bar, and TRTfFadiga when it was released.

All participants received identical verbal instructions that were read from a standardized script by the same trained examiner to ensure uniformity of delivery. Before the experimental block, the participants completed a 10-trial practice. The task was performed in a sound-attenuated room with fixed screen distance (60cm) and controlled illumination. Stimuli appeared at randomized intervals (1.5-6.5s), and participants were instructed to respond as quickly and accurately as possible by pressing a single button with the dominant hand. No online feedback was provided; only a summary of the mean reaction times was displayed at the end of each block. Trials with anticipatory (<100ms) or delayed (>1000ms) responses were automatically discarded. These procedures ensured reproducibility and minimized behavioral variability that could influence the EEG responses.

#### Brain activation - electroencephalogram

**Figure 2:**
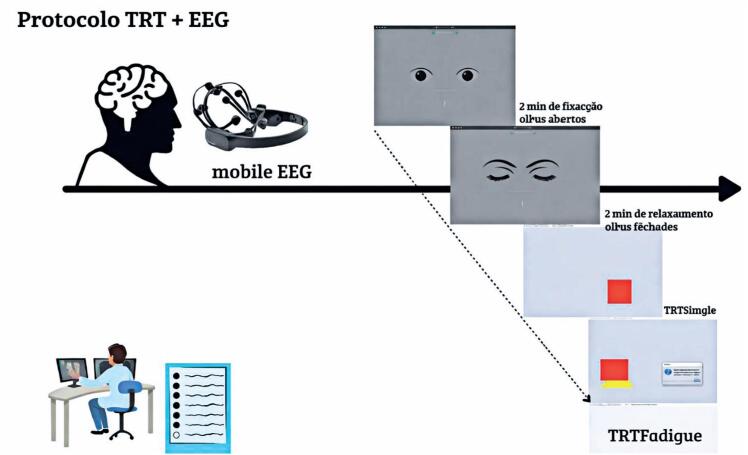
Electroencephalography preparation protocol for the motor reaction time test

Electroencephalography was performed using the EMOTIV Epoc+ 14 channel wireless EEG instrument (Copyright© EMOTIV Inc., San Francisco, USA) during the motor reaction time task as shown in figure 2.

**Figure 3:**
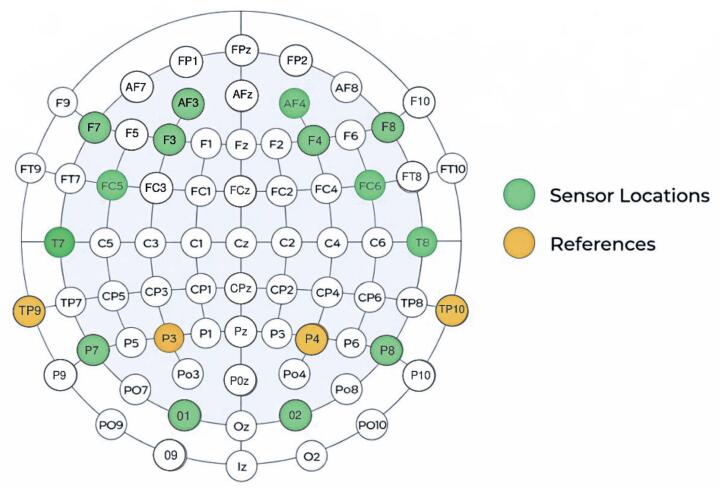
Location of electrodes, international system 10-20

The EMOTIV EPOC+ is a high-resolution portable EEG device equipped with 14 electrodes for data acquisition (AF3, F7, F3, FC5, T7, P7, O1, O2, P8, T8, FC6, F4, F8, and AF4) and two reference electrodes (P3 and P4).^(45-47)^ The EEG allows real-time monitoring of neural activity in the brain with millisecond precision, sensitively detecting small variations in neural oscillations.^(48)^ The positioning of the electrodes was guided by the international 10-20 system as illustrated in figure 3, which ensures adequate coverage of the frontal, prefrontal, temporal, parietal and occipital lobes.

The device is simple to set up and use; the sensors are simply wetted with saline solution, installed on the EEG, and the position of the reference electrode is adjusted until the indicator light reaches 100%. The hair was moved aside to expose the scalp, where 14 channels received EEG signals for independent reading. The patients performed the following procedure to establish the EEG baseline: (1) 3 s of preparation with countdown; (2) 15 s of recording with eyes open, accompanied by countdown; (3) 3 s of preparation with countdown; and (4) 15 s of recording with eyes closed and countdown (Figure 2).

Data analysis was performed by initially processing the raw data using TestBench software (Copyright© EMOTIV Inc., San Francisco, USA). Subsequently, the data were subjected to signal analysis and filtering using MATLAB R2017 software (MathWorks, Inc., Natick, Massachusetts, USA). Based on the EEG signal, the level of arousal was determined by calculating the ratio of beta (β) (12-28 Hz) and alpha (α) (8-12 Hz) brain waves. Only the electrodes of the frontal and prefrontal cortices were considered for data analysis: AF3, AF4, F3, F4, FC5, FC6, F7, and F8. Beta waves (β) are associated with an alert or excited mental state, while alpha waves (α) are more dominant in a relaxed state. Alpha activity (α) has also been associated with brain inactivation.^(49,50)^ Thus, the beta/alpha ratio is a reasonable indicator of an individual's state of arousal.

EEG preprocessing and trial classification were performed independently by two raters who were blinded to group allocation and behavioral data. Agreement between the raters was high (Cohen's κ=0.87; ICC=0.92, 95% CI 0.88-0.95), and discrepancies were resolved by consensus with a third blinded evaluator. This double-blind procedure reduced the subjective bias in data interpretation.

### Statistical analysis

Data normality was analyzed using the Shapiro-Wilk test. A descriptive presentation was made using measures of central tendency (mean) and variability (standard deviation) for continuous variables and measures of frequency and percentage for categorical variables. One-way analysis of variance (ANOVA) with Tukey's post-hoc analysis was used to analyze the differences between volunteers stratified by sarcopenia phenotype, following the diagnostic criteria for sarcopenia proposed by the EWGSOP. Pearson's product correlation (r) was calculated to assess the relationship between discriminative variables, health characteristics, functional abilities, and motor reaction time. A correlation of r<0.3 was considered ‘weak,’ 0.31-0.60 was considered ‘moderate,’ and >0.6 was considered ‘strong.’ The Benjamini-Hochberg false discovery rate (FDR; q=0.05) correction was applied to all EEG-related tests to control for multiple comparisons across electrodes and frequency bands. All analyses were performed using IBM® SPSS® Statistics software (version 26.0), with a significance level of 5% (α=0.05).

## RESULTS

### Descriptive data

The groups were homogeneous in age and BMI. However, significant differences were observed in body weight, body fat, and body mass between the control and sarcopenic groups (Table 1).

**Table 1:** Descriptive and analysis of anthropometric and body composition variables according to sarcopenia status

Mean (Standard deviation)				95% CI	p value
non-sarcopenic			72.10 (±7.57)	68.46 - 75.75	
pre-sarcopenic			71.74 (±10.73)	66.57 - 76.91	0.733
Age (years)					
		sarcopenic	70.19 (±5.71)	67.59 - 72.79	
		Total	71.31 (±8.09)	69.20 - 73.41	
		non-sarcopenic	58.84 (±6.12)	55.89 - 61.79	
		pre-sarcopenic	63.85 (±9.67)	59.18 - 68.51	0.033
Weight (kg)					
		sarcopenic	67.74 (±13.71)	61.50 - 73.98	
		Total	63.62 (±10.91)	60.78 - 66.46	
		non-sarcopenic	24.90 (±2.28)	23.80 - 26.00	
		pre-sarcopenic	26.24 (±4.01)	24.31 - 28.17	0.277
BMI (kg/m2)					
		sarcopenic	26.98 (±5.27)	24.58 - 29.38	
		Total	26.07 (±4.11)	25.00 - 27.14	
		non-sarcopenic	34.37 (±3.50)	32.68 - 36.06	
		pre-sarcopenic	38.90 (±5.45)	35.51 - 42.30	0.022
Fat (%/kg)					
		sarcopenic	39.21 (±5.85)	36.39 - 42.03	
		Total	37.54 (±6.20)	35.93 - 39.16	
		non-sarcopenic	27.74 (±1.41)	27.06 - 28.42	
		pre-sarcopenic	25.10 (±3.08)	23.69 - 26.50	0.009
Muscle (%/kg)					
		sarcopenic	23.89 (±2.28)	22.79 - 24.99	
		Total	25.88 (±2.68)	25.18 - 26.58	

The correlations between body structure and function, motor reaction time (MRT), and HGS were significantly different between the groups. The MRT significantly differed between the control and sarcopenic groups. Table 2 shows weak, moderate, and strong intragroup correlations. Body structure and function variables were divided into muscle percentage, fat percentage, muscle strength, and the 30-s sit-to-stand test.

**Table 2:** Intragroup correlations between motor reaction time and body structure, function, personal factors, and functional skills according to sarcopenia status

	Non-sarcopenic			Pre-sarcopenic			Sarcopenic		
	MRT	TRTS	TRTF	MRT	TRTS	TRTF	MRT	TRTS	TRTF
Body structure and function									
Muscle (%/kg)	-0.235	-0.199	-0.684	-0.350	-0.891	-0.251	-	-	-
Fat (%/kg)	-	-	-	-0.606	0.644	0.360	-	-	-
Muscle strength (kg/f)	-0.777	-0.611	-0.719	-0.325	-0.541	0.275	-	-	-
30-Second Sit to Stand (repetitions)	-0.565	-0.337	-0.756	-0.851	-0.820	0.165	-	-	-
Personal factor									
FES-I (score)	0.743	0.319	0.643	0.621	0.900	0.034	-	-	-
Functional skills									
TUG (seconds)	0.991	0.904	0.112	0.722	0.763	0.361	0.408	0.032	0.765
4-Stage Balance test (score)	-0.728	-0.968	-0.352	-0.220	-0.143	-0.901	0.532	0.231	0.482
Gait speed (m/s)	-0.118	-0.526	-0.975	-0.355	-0.142	-0.490	-0.496	-0.050	-0.584
Step length (m)	-0.988	-0.977	-0.352	-0.620	-0.054	-0.548	-0.675	-0.509	-0.238

Pearson correlation test, considering significant p<0.05, weak correlations with r<0.3, moderate correlations with r=0.3 to 0.6, and strong correlations with r >0.6.

A: non-sarcopenic; B: pre-sarcopenic; C: sarcopenic; FES-I: Falls Efficacy Scale International; HGS: handgrip strength; MRT: motor reaction time; TRTS: simple motor reaction time; TRTF: motor reaction time with fatigue; TUG: Timed Up and Go test; - the absence of significant correlation (p≤0.05).

Muscle percentage was assessed in terms of body composition and revealed weak and moderate indirect correlations in the Control Group and moderate and strong correlations in the pre-sarcopenic group, indicating decreased MRT with increased muscle percentage. Conversely, fat percentage was assessed in terms of body composition and revealed no correlation in the Control Group. However, a moderate direct correlation was observed in the pre-sarcopenic group, indicating that the higher the fat percentage, the slower the motor response of older women to the stimulus. Muscle strength showed strong indirect correlations in the Control Group and moderate indirect correlations in the pre-sarcopenia group, suggesting that the greater the muscle strength of older women, the faster their motor response to stimuli.

The 30-second sit-to-stand test showed moderate and strong indirect correlations between the groups, highlighting that a greater number of repetitions in the test meant that older women had a faster MRT.

### Correlation between personal factors and motor reaction time

Personal factors were based on the FES-I, which assesses the participant's level of concern with falls while performing each activity of daily living. The findings revealed that the FES-I had moderate and strong direct correlations between the groups. This indicates that higher FES-I scores were related to slower MRT.

In the sarcopenic group, no significant correlations were observed between MRT and the percentages of muscle and fat, muscle strength, the 30-second sit-to- stand test, and FES-I score. This indicates that the set of body structure and function variables and personal factors do not impact or are not related to sarcopenia in older women during activity.

### Correlation between functional skills and motor reaction time

Functional skills assessed in this study included TUG, the 4-stage balance test, gait speed, and step length. Strong direct correlations were found in the TUG test in the control and pre-sarcopenic groups, while moderate direct correlations were observed in the sarcopenic group. This indicates that the longer the TUG test, the faster the MRT in older women.

In the 4-stage balance test, moderate and strong indirect correlations were observed in the Control Group, whereas weak indirect correlations were found in the pre-sarcopenia group. This suggests that the more positions maintained for 10 s in the test, the faster the MRT of the older women. In the sarcopenia group, moderate direct correlations were observed in suggesting that the participants had slower MRT compared to that of those in the other groups. This means that the more positions the participant maintained in the 4-stage balance test, the more balance they had and the slower their MRT.

Moderate and strong indirect correlations were observed for the gait speed between the groups, indicating that the greater the gait speed, the faster the MRT. The findings also indicated moderate and strong indirect correlations in step length between the groups, demonstrating that the greater the step length, the faster the MRT. Sarcopenia slows down in older women and therefore decreases their gait speed and step length, increasing their MRT during the task.

### Cortical excitation with EEG and sarcopenia

Table 3 presents the distribution of means and standard deviations of the cortical excitation analysis through α and β factors with EEG. The α wave is responsible for rest, sleep, and depression in cortical activity. The β wave is guided by voluntary or intentional acts, i.e., voluntary motor control.

**Table 3:** Cortical excitation in Alpha and Beta bands across EEG channels in non-sarcopenic, pre-sarcopenic, and sarcopenic groups

Channels	Non-sarcopenic		Pre-sarcopenic		Sarcopenic		Alpha p value	Beta pvalue
	Alpha	Beta	Alpha	Beta	Alpha	Beta		
AF3	6.26±1.25	6.74±1.95	4.12±1.02	7.82±1.62	6.32±1.18	6.02±1.74	0.042	0.031
F3	5.18±2.25	8.08±1.89	3.21±0.95	10.25±1.26	6.21±1.89	7.29±1.71	0.018	0.015
F7	3.25±0.89	3.21±1.03	2.79±0.51	4.81±1.28	4.02±1.11	3.25±1.29	0.031	0.029
FC5	1.23±0.63	3.31±1.06	1.09±0.31	6.71±1.29	1.91±0.13	3.74±1.06	0.042	0.018
FC6	1.17±0.98	3.28±0.61	1.06±0.54	6.89±0.61	2.03±0.74	3.73±0.49	0.026	0.018
F4	5.25±0.54	7.76±1.25	3.48±0.71	10.96±1.56	6.13±0.71	7.31±1.21	0.019	0.021
F8	3.29±1.52	3.63±1.01	2.19±1.61	4.72±1.31	4.37±1.19	3.98±1.36	0.034	0.031
AF4	6.18±1.10	7.14±1.89	3.98±1.98	8.26±1.15	6.89±1.08	6.77±1.71	0.039	0.030

AF3: left anterior frontal region; F3 and F7: left frontal regions; FC5: left frontocentral region; FC6: right frontocentral region; F4 and F8: right frontal region; AF4: right anterofrontal region; SD: standard deviation; unit: dB/Hz.

**Figure 4:**
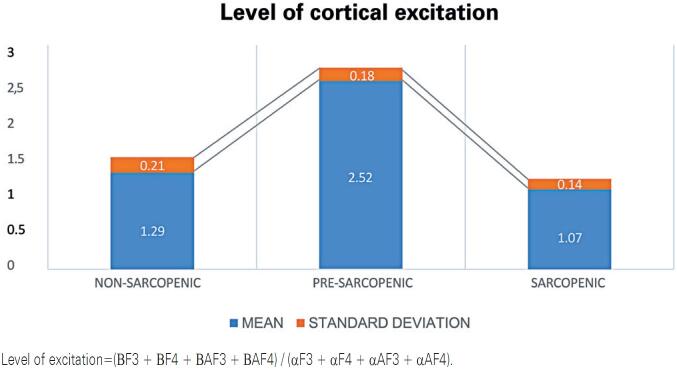
Distribution of intragroup means and standard deviations of the level of cortical excitation

Four of the eight channels tested stood out and were responsible for motor planning and processing: F3, FC5, FC6, and F4. The mean values were higher in the pre-sarcopenia group compared to those in the other groups. Table 4 presents the level of cortical excitation determined by the ratio between beta (12-28 Hz) and alpha (8-12 Hz) brain waves. Figure 4 also highlights the mean cortical excitation value, which was almost twice as high in older women with pre-sarcopenia as in the other groups, demonstrating that these women had a rather high and significant cortical excitation level (p>0.001), which deserves discussion.

**Table 4:** Cortical Excitation Index

	Level of Cortical Excitation			
	Mean	Standard Deviation	Confidence Interval	p value
Non-Sarcopenic	1.29	0.21	0.88-1.12	
Pre-Sarcopenic	2.52	0.18	1.91-2.09	<0.001
Sarcopenic	1.07	0.14	0.93-1.08	

Level of excitation=(ΒF3 + ΒF4 + ΒAF3 + ΒAF4) / (αF3 + αF4 + αAF3 + αAF4).

Level of excitation=(ΒF3 + ΒF4 + ΒAF3 + ΒAF4) / (αF3 + αF4 + αAF3 + αAF4).

## DISCUSSION

The findings of this study indicate a depression of cortical excitation assessed with EEG in older women with sarcopenia and a statistically significant difference (p<0.001) in the level of cortical excitation in older women with pre-sarcopenia compared to that in the Control Group. Although cortical excitation changes might also reflect attentional or sensory processing, our analyses focused on motor-related cortical areas (F3, FC5, FC6, F4) and on alpha and beta rhythms classically associated with voluntary motor preparation and execution. The temporal alignment of EEG epochs to response onset, together with the predominance of contralateral activation, supports the interpretation of motor-related rather than purely sensory cortical activity.

Studies on aging in the human brain have shown that the regulation of dopamine action is significantly reduced in old age and, according to the literature,^(51)^ the increased reaction time in older adults is related to the loss of dopamine receptors. This change occurs because of structural degradation, including neuronal loss, reduction in neuroreceptor sites, and a decline in transporter molecules.^(52)^ Older people have higher rates of cortical hypoexcitability^(53)^ and progressive degeneration of dopaminergic neurons in the midbrain is associated with deficits in the initiation, speed, and flow of voluntary movement.^(54)^

Older women with pre-sarcopenia had a significantly higher level of cortical excitation as the body was undergoing adaptations and adjustments due to the loss of muscle percentage, which makes the motor conduction time and cortical activation faster. The Scaffolding Theory of Aging and Cognition (STAC) postulates that increased brain activity with age, especially in the prefrontal cortex, is a compensatory mechanism caused by brain reorganization in response to age-related declines in neural structures and functioning.^(55)^ For high-demand tasks that require processing beyond the level of available neural resources, older brains may use compensatory mechanisms to improve age-related declines in the temporal processing of sensory stimuli when producing movements.^(56)^

Significant differences were found between the control and pre-sarcopenic groups in the correlation between the MRT and muscle percentage, fat percentage, HGS, leg strength, and resistance assessed using the 30-second sit-to-stand test.

In the pre-sarcopenia group, strength showed a favorable correlation with MRTS and MRTF reduction. There was a strong correlation with the 30-second sit- to-stand test and a moderate correlation with HGS. This difference may have occurred because the 30-second sit-to-stand test requires not only isometric strength (as in HGS) but also concentric and eccentric strength.

The difference in HGS was significant between the groups because muscular hypotrophy is a characteristic of aging and associated with significantly impaired function (such as slowness of movement and muscle weakness), consequently impairing the exchange of sensorimotor information.^(57)^

In the healthy aging process, muscle regeneration pathways are activated in healthy older people without sarcopenia but can be dysregulated or simply overloaded in sarcopenic older adults.^(58)^ Muscles may be damaged, and because of their inability to regenerate when injured, they become slower in performing tasks, thus affecting older people's functioning.

These data corroborate the present research, which found a positive correlation between muscle percentage and MRT, showing that the lower this percentage, the slower the MRT, as well as muscle strength and the 30-second sit-to-stand test; that is, the lower the strength and the fewer the repetitions in the test, the slower the response to the stimulus.

Our findings suggest that older women with sarcopenia need a long motor processing time to achieve a good balance score; that is, they slow down as a strategy to make functional balance adjustments. The correlations of MRT with fear of falling (assessed with FES-I) and static balance (assessed with the 4-stage balance test) were significantly different between the groups. This can be explained by the aging process, which leads to the loss of muscle mass, muscle strength, and reduced mobility, which are factors directly related to the risk of falling. In addition, according to a systematic review, older people with sarcopenia have a 34-91% significantly greater risk of falling.^(30)^

This study did not find significant correlations between MRT, body structure and function, or personal factors in older women with sarcopenia. A possible explanation is that such women supposedly have an impaired pyramidal system and worsened brain-muscle communication. In other words, a neuromuscular system that does not function well in sarcopenia may be due to impaired corticospinal interactions, which, in turn, can cause the muscle microenvironment to lose muscle mass, strength, and functionality.^(58)^

The correlations between gait speed, step length, and MRT were significantly different between the groups, which can be explained by the different changes in gait associated with aging, such as slowness and greater gait variability.^(59)^

Older people have greater metabolic costs in terms of gait, mainly owing to the loss of strength and muscle mass. Furthermore, a slower gait speed in older adults is a form of adaptation that occurs in response to various muscle-related physiological changes.^(60)^ This is in line with the findings of this study, in which slowed down sarcopenic older people had decreased gait speed and step length, leading to a longer MRT.

The data in this study show that the higher the gait speed, the lower the MRTF. Hence, as older women can remain attentive to the task, their "sixth functional vital sign" (*i.e*., the gait speed) improves – as described in the literature that an association was also found between gait speed and the attention level.^(61)^

According to these data, pre-sarcopenia seems to be the ideal time to carry out interventions, as the brain is attentive to dysfunction and is receptive to stimuli. These results suggest that resistance exercise is a promising strategy to ensure brain health and prevent neurological diseases. They may also shape the frontal lobe, which is accompanied by an improvement in executive function. Thus, exercise is a promising approach for ensuring brain health.^(62)^

Future clinical research should focus on the application of these findings to the design of new therapeutic interventions. What is emphasized here is that in the future, further external relationships may be included in studies correlating brain activation patterns (EEG) in sarcopenic older women and MRT. An intervention protocol may also be developed for older women with pre-sarcopenia, thus helping prevent and treat sarcopenia, further understanding its complexity and relationship with brain health, and better interpreting it for clinical practice.

Notably, the use of scalp EEG inherently limits spatial precision. Source reconstruction analyses were not performed in this study; therefore, anatomical interpretations should be regarded as topographical rather than region specific. Nevertheless, the Emotiv EPOC+ system used here follows the international 10-20 electrode placement standard, ensuring consistent anatomical correspondence across participants. Previous validation studies confirmed the reliability of this montage for cortical mapping and alpha- and beta-band analyses of motor processing.^(63-65)^ These characteristics support the reproducibility and physiological plausibility of topographical findings.

This study has some limitations that should be considered. The inclusion of only older women may limit the generalizability of the results to other populations. Future studies should include more diverse samples and incorporate advanced neuroimaging techniques to provide a more comprehensive understanding of the neural mechanisms underlying sarcopenia.

## CONCLUSION

Sarcopenia is not only a result of natural aging processes but is also directly related to central changes in neurodegenerative processes. EEG has proven effective in assessing these changes, as observed in the cortical activity of older women.

According to the results of this study, the pre-sarcopenia phase is the ideal time to initiate neurotherapeutic interventions aimed at mitigating the adverse consequences of sarcopenia, as the brain is most sensitive to dysfunction and most receptive to stimuli at this stage.

Variables related to body structure, body function, and personal factors did not appear to affect motor reaction time to activity in older women with sarcopenia, thus highlighting the complexity of this condition and the need for a holistic approach.

Based on these findings, this study may contribute to targeting strategic points for interventions and more in-depth investigations into brain behavior and neurodegenerative processes in this specific population, highlighting the importance of integrated approaches that consider a variety of physical, emotional, and social factors.

## Data Availability

The datasets generated and/or analyzed during the current study are available from the corresponding author upon reasonable request.
